# Supplementary material for: Centimeter-scale nanomechanical resonators with low dissipation
Source: Nat Commun. 2024 May 18;15:4255. doi: 10.1038/s41467-024-48183-7 (PMC11102468; doi:10.1038/s41467-024-48183-7)
Supplement: Supplementary file 1 — Supplementary Information [file 41467_2024_48183_MOESM1_ESM.pdf]

Supplementary Information: Centimeter-scale nanomechanical resonators with low dissipation

Andrea Cupertino,[1,*] Dongil Shin,[2,3,*] Leo Guo,[4] Peter G. Steeneken,[2,5] Miguel A. Bessa,[6,†] and Richard A. Norte[2,5,‡]

[1]*Department of Precision and Microsystems Engineering, Delft University of Technology, Mekelweg 2, 2628CD, Delft, The Netherlands*
[2]*Department of Precision and Microsystems Engineering, Delft University of Technology, Mekelweg 2, 2628 CD, Delft, The Netherlands*
[3]*Department of Materials Science and Engineering, Delft University of Technology, Mekelweg 2, 2628 CD, Delft, The Netherlands*
[4]*Department of Microelectronics, Delft University of Technology, Mekelweg 2, 2628 CD, Delft, The Netherlands*
[5]*Kavli Institute of Nanoscience, Department of Quantum Nanoscience, Delft University of Technology, Lorentzweg 1, 2628 CJ, Delft, The Netherlands*
[6]*School of Engineering, Brown University, 184 Hope St., Providence, RI, 02912, USA*

CONTENTS

1. Gas damping 2
2. Variation of quality factor as function of thickness 3
3. Stitching errors in electron beam lithography 3
4. Photoresist cracking at cryogenic temperature 5
5. Beams collapsing 6
6. Optothermal effects on the measured Q factor 7
7. Multi-fidelity Bayesian optimization 8
8. Multi-fidelity Bayesian optimization initial random points dependency 9
9. PnC beam resonator's design parameters 9
10. Quality factors of additional devices 10
11. Ultra High Vacuum Setup 11

References 12

* These authors contributed equally: Andrea Cupertino, Dongil Shin.
† email:miguel_bessa@brown.edu
‡ email:r.a.norte@tudelft.nl

1. Gas damping

Room temperature quality factors of resonators fabricated from highly pre-stressed films are typically proportional to the resonator's aspect ratio ($Q \propto L/t$). Thinner films tend to exhibit a lower intrinsic quality, which makes increasing the length (L) a favorable design strategy. If a mechanical mode is only limited by bending losses, designs scaled up in L should show higher quality factor. To utilize this, we strategically aim for the longest possible multi-centimeter scale designs as shown in Figure S1. Nevertheless, we encounter a trade-off: as resonators grow beyond 3 cm, they are more prone to gas damping — a result of interactions with gas molecules — that increases with lower resonance frequencies, and higher aspect-ratio mechanical modes. Thus longer designs require lower vacuums to evade gas damping Equation S5. With a turbo and roughing pump, the lowest achievable pressure in our UHV chamber is 10^{-9} mbar (Figure S14), limiting our nanostrings lengths to 3 cm. With more advanced pumping schemes, it is possible to achieve pressures of 10^{-11} mbar[18] and further increase resonators' lengths beyond 3 cm to achieve quality factors beyond 10 billion. This section discusses the experiments performed to confirm this boundary.

Gas damping originates by the interaction between the moving surface of the resonators with the gas molecules around it and it often dominates the extrinsic contributions at ambient condition. The nature of this loss mechanism depends on the amount of gas molecules surrounding the resonators, hence the pressure of the gas and the gas composition compared to the resonator's dimension. It can therefore be suppressed by reducing the pressure value at which the resonators operate.

To this end, we first need to evaluate the regime in which the resonator is operating and the consequent dominant gas damping mechanisms. This can be done by calculating the Knudsen number (K_n), describing the ratio of the gas mean free path length (λ_f) to the representative physical length scale of the resonator (L_r)[19]:

$$K_n = \frac{\lambda_f}{L_r} \tag{S1}$$

The gas mean free path length can then be calculated by the following equation:

$$\lambda_f = \frac{k_B T}{\sqrt{2}\pi d_{gas}^2} \frac{1}{p} \tag{S2}$$

where k_B is the Boltzmann constant, T is temperature, d_{gas} is the diameter of the gas particles, and p is the gas pressure. Atmospheric air possesses a mean free path of approximately 70 nm, several orders of magnitude lower than the representative physical lengths of the nanomechanical resonators hereby developed. It follows that at the ambient condition the resonators are dominated by viscous damping.

By decreasing the pressure, we enter the ballistic regime where the resonator dimensions become compared or smaller than the gas mean free path (Equation S2), hence K_n becomes larger than unity. In this regime, the quality factor scales with the

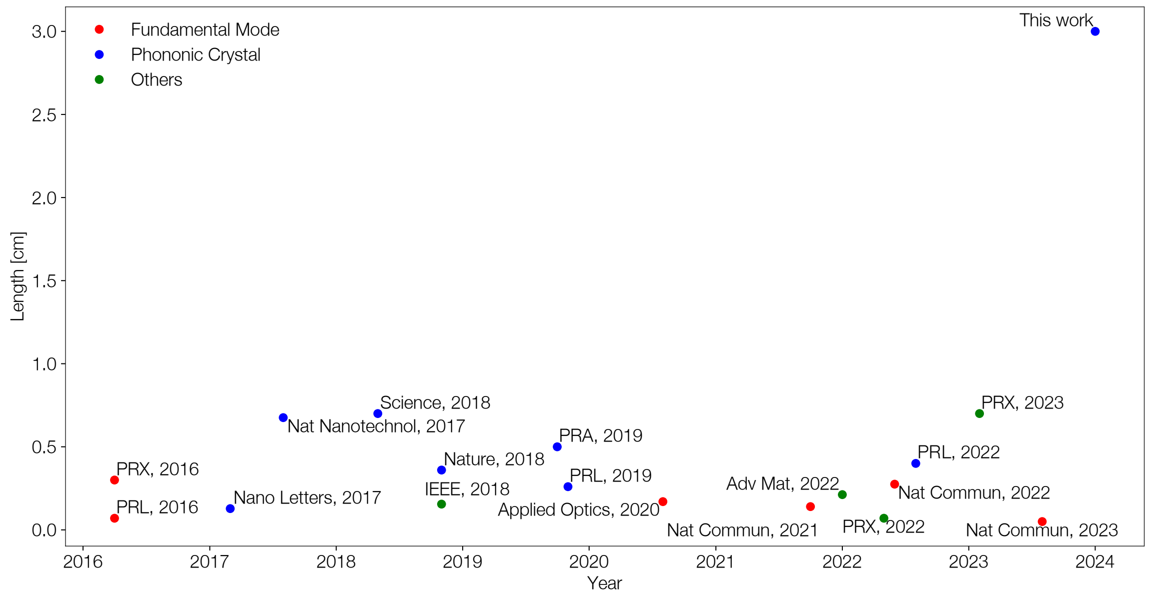

FIG. S1. Room temperature quality factors of resonators fabricated from highly pre-stressed films are proportional to a resonators lateral size. Here we show a variety of high-Q room temperature mechanical resonators over the last years plotted in accordance to their lateral size vs. publication year. The colors indicate what type of mechanical mode is used. The datapoints in the plot, from left to right, correspond to[1–17]

pressure until gas damping becomes negligible compared to other sources of losses. The quality factor in this regime can be calculated from an energy transfer model as[20]

$$Q_{free} = \frac{\rho t \omega}{p}\sqrt{\frac{\pi R T}{32M}} \tag{S3}$$

where ρ is density, t is the resonators thickness, ω is the resonance frequency, R is the molar gas constant, T is the temperature, and M is the molar mass of the gas ($28.97\,\mathrm{g\,mol^{-1}}$ for air). If there is another surface in close proximity to the resonator, contributions from squeeze-film damping need to be considered and the quality factor can be calculated from

$$Q_{sq} = 16\pi\frac{d}{L}Q_{free} \tag{S4}$$

where d is the gap height between the resonator and nearby surface, and L is the resonator length. The total quality factor is then given by

$$Q_{tot}(p) = \left(\frac{1}{Q_{int}} + \frac{c_0}{Q_{sq}(p)}\right)^{-1} \tag{S5}$$

where Q_{int} is the intrinsic quality factor when gas damping becomes negligible and c_0 is an experimental scaling factor[21]. It is important to notice that gas damping is not the only source of extrinsic loss. Specifically radiation loss, caused by the hard clamping of the resonator to the supporting substrate, has been reported to play a critical role. However, the nanomechanical resonators here developed employ a PnC to isolate the mechanical mode from environmental noise. This highly suppresses radiation loss as previously reported for similar designs[4]. We therefore consider the quality factor limited only by intrinsic contributions once gas damping becomes negligible. The intrinsic losses are addressed in the main text.

To quantify the effect of gas damping we performed ringdown measurements of the 3 cm nanomechanical resonator at different pressure levels and extracted the resulting quality factor from each dataset. The data points are then fitted using Equation S5 with c_0 and Q_{int} as fitting parameters. The result in Fig. S2 demonstrates that gas damping becomes negligible (less than 5%) for pressure levels approaching 1×10^{-9} mbar. The extracted quality factor equals the intrinsic value of 3.42 billion for this specific resonator. We therefore carried out all the measurements reported in the main text at this pressure level.

While the specific resonator employed for the pressure study hereby described is not the same device used for the results reported in Fig. 4 of the main text, the geometrical dimensions are equal. We can hence assume a similar gas damping behavior. Moreover Equation S5 has been developed for a beam with a uniform width, while the width of our resonators varies along its entire length due to the applied tapering and the PnC. One then has to resort to numerical simulations for an accurate calculation of the quality factor at every pressure level. However, the purpose of this study is to simply find the vacuum requirements to extract the intrinsic quality factor of the fabricated resonators not limited by gas damping, rather than accurately capturing the gas damping behavior. For this purpose, Equation S5 provides an accurate lower estimate.

2. Variation of quality factor as function of thickness

The predicted Q factor of the optimized centimeter-scale nanomechanical resonators is 10 billion, while the fabricated structures show a Q factor of 6.6 billion. We believe the observed difference is caused by difficulties to dissipate heat during the undercut process caused by the high-aspect-ratios. This results in different thicknesses and dimensions from edge to center along the beam of the fabricated nanostructures, with a significant effect on the measured Q factor.

In this section, we investigate the case for which the thickness of the Si_3N_4 layer is uniformly 20 nm larger than the expected thickness, a value compatible with the etch settings employed during the undercut in view of the Si_3N_4 etch rate in SF_6 plasma etching and the temperature of the process.

This thickness variation results in a reduction of the quality factor from over 1 billion at 50 nm to 6.9×10^9 at 70 nm, as shown in Fig. S3a. This value is in good agreement with the value experimentally measured (Fig. 4a of the main text) of 6.6×10^9. On the contrary, the thickness has a negligible effect on the resonance frequency value as displayed in Fig. S3b, where f_0 is the resonance frequency obtained for the 30 nm-thick resonator, corresponding to 213.323 kHz. This value agrees with experiments to around 1%, further confirming the high-fidelity between simulations and experiments.

3. Stitching errors in electron beam lithography

A pattern in electron beam lithography is generated by deflecting the electron beam based on the desired shape. However, due to the finite sweep range of the deflector system, the writing field cannot exceed an area of typically 100 µm - 1 mm. The

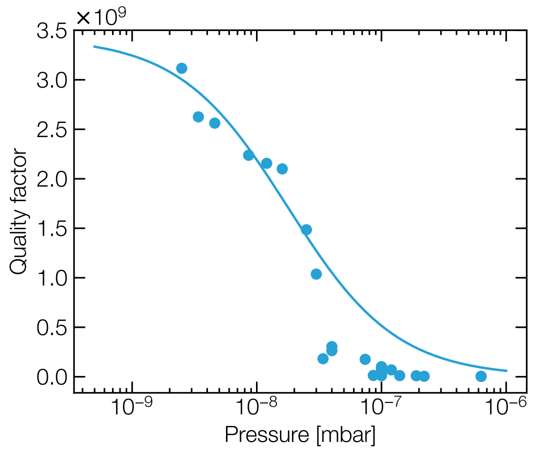

FIG. S2. Measured quality factor as a function of pressure for a 3 cm optimized nanomechanical resonator. The blue line shows the fit, and the blue points indicate the experimental data.

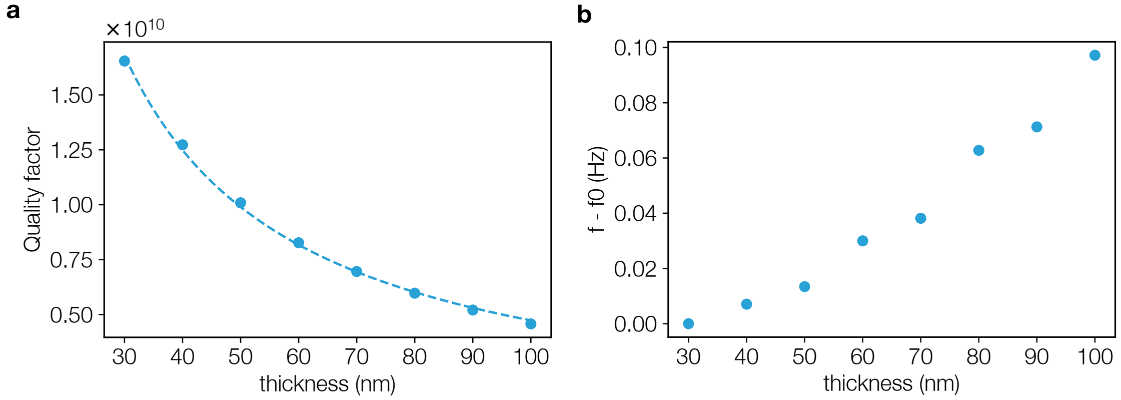

FIG. S3. **a**, Quality factor as a function of the resonator thickness. **b**, Difference of the eigenfrequency value as a function of the resonator thickness. The data are obtained by finite element analysis by varying the thickness of the optimized design described in the main text. f_0 is the resonance frequency obtained for the 30 nm-thick resonator, corresponding to 213.323 kHz.

maximum writing field for the Raith EBPG 5200 machine at Kavli Nanolab in Delft is 1040 µm. Large patterns need therefore to be stitched together by moving the sample on a stage. Misalignment between multiple writing fields might then arise due to miscalibration and thermal drift among other reasons. The consequent stitching errors have significant effects on the obtained shape[22]. This is particularly relevant for high stress Si_3N_4, where a misalignment of the order of nanometers can lead to stress concentration and rupture of the fragile suspended structure.

The centimeter-scale nanomechanical resonators hereby developed require 30 writing fields to be correctly patterned by electron beam lithography. In order to accurately transfer the desired shape, we employed an electron beam with an estimated spot size equal to 18 nm and a spacing of 5 nm. The fine resolution results in a long writing time exceeding one hour, which increases the likelihood of misalignment and consequent stitching errors. For most of the exposed devices we in fact observed an incorrect patterning at the boundary of every writing field as shown in the dark-field microscope picture in Fig. S4a. Figure S4b provides a picture with higher magnification of the same structure which clearly shows a discontinuity of the written pattern.

In order to mitigate the observed stitching errors we focused first on reducing the long writing time. We did so by performing multiple exposures with different resolutions. The most critical features were exposed with a fine electron beam and high resolution, while a coarse electron beam with lower resolution was employed for the remaining areas of the pattern. As a result, the total writing time for a single 3 cm nanomechanical resonator was reduced down to 10 minutes without affecting the accuracy of the desired pattern. This was effective to eliminate the systematic misalignment previously present at every writing field boundary (Fig. S4a), however, some stitching errors could still be observed in a random manner, varying from run to run.

To this end, we changed the dose at different positions to take into account possible underdose at the boundary between writing fields and overlapped each writing field with the nearby one for 100 nm. This resulted in a correct exposure of the desired pattern,

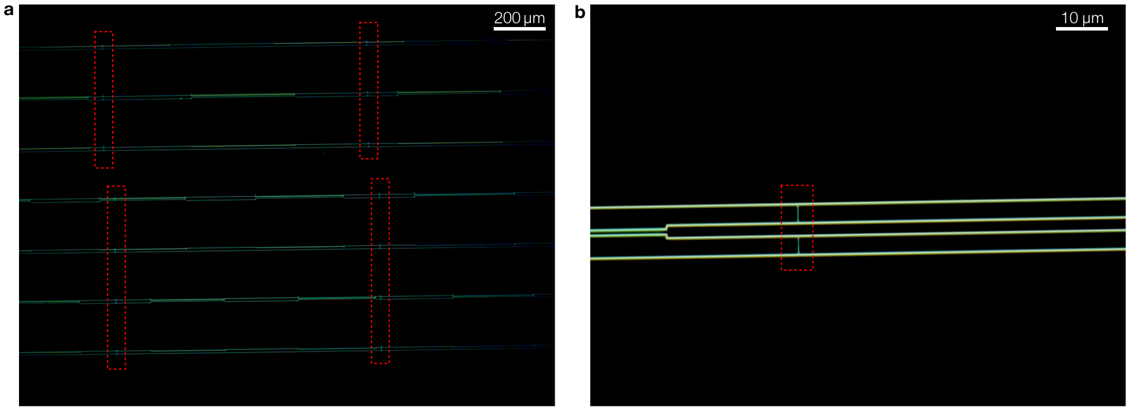

FIG. S4. **a**, Dark-field microscope picture of a pattern with multiple structures exceeding the writing field of the electron beam lithography. **b**, Zoom-in dark-field microscope picture of a single structure at the boundary between two writing fields. The dotted red square highlights the misalignment observed between consecutive writing fields. The pictures are taken after developing the exposed e-beam resist.

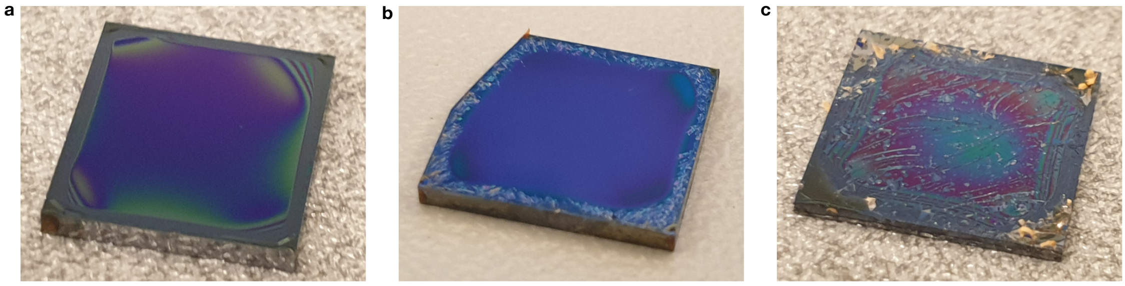

FIG. S5. Photograph of silicon chip with photoresist spin coated on top before and after cryo-DRIE etching. **a**, Device before the cryo-DRIE step with uniform photoresist layer **b, c**, Devices with different photoresist layer after cryo-DRIE etching.

without any noticeable stitching error, and an accurate transfer of the centimeter-scale nanoresonators geometry.

4. Photoresist cracking at cryogenic temperature

Cryogenic deep reactive ion etching (DRIE) is employed in the fabrication of the centimeter-scale nanoresonators to increase the gap separating the suspended Si_3N_4 from the supporting Si substrate. Contrary to other deep silicon etching techniques as the Bosch process, cryo-DRIE is known to result in a smoother sidewall[23], and it does not leave carbon residues on the sidewall. In fact, the oxide passivation layer created to anisotropically etch Si dissolves at high temperatures. At the same time, its main drawback is the vulnerability of photoresists to cracking at low temperatures.

Previous studies found that cracking depends on the layer thickness and the specific photoresist material. The thin photoresist of less than 1.5 µm are in fact reported to be free from cracking. Moreover, materials with a high degree of cross-linking and high mechanical strength do not suffer from cracking even for larger thickness. We then studied the effect of cryo-DRIE on the photoresist SU1813, spin coated on Si_3N_4 chips with thickness varying from 1.4 µm to 2 µm, before being exposed to the fluorine-based cryo-DRIE. The thickness is varied within an interval which provides enough material to obtain the desired gap size due to the photoresist selectivity. The thicker photoresist (2 µm) shows cracks over the entire surface (Fig. S5c), while in the thinner photoresist (1.4 µm) the cracks are localized at the outer area of the chip (Fig. S5b), in good agreement with the expected behavior. The cracks for the outer area of the thin photoresists are most likely caused by the non-uniform thickness at the edge, as Fig. S5a shows. We repeated the analysis for a second photoresist, AZ5214, without noticing any significant difference.

We therefore focused on a photoresist thickness of 1.4 µm (Fig. S5b) in which the central area remains intact. Despite most of the surface being free from cracking, we observed that some cracks might propagate from the outer area towards the center with fatal consequences on the nanoresonators. Figure S6a shows an example where the cracks propagate toward the nanomechanical

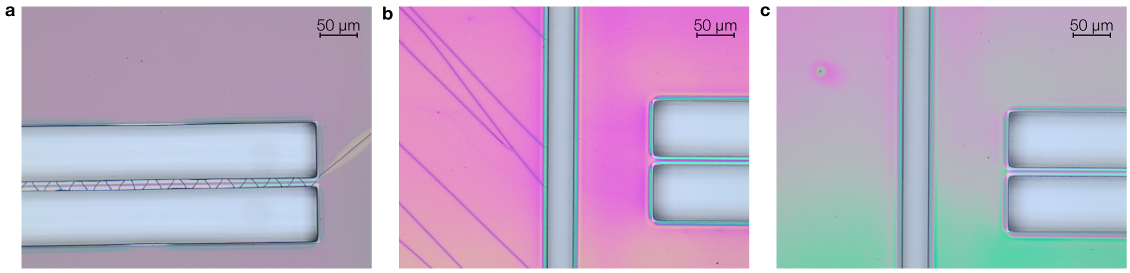

FIG. S6. Optical microscope pictures of the photoresist layer after cryo-DRIE. **a**, Pattern without outer ring in which the cracks propagate toward the centimeter-scale nanoresonator. **b-c**, Pattern with the outer ring which protects the centimeter-scale nanoresonator from the cracks.

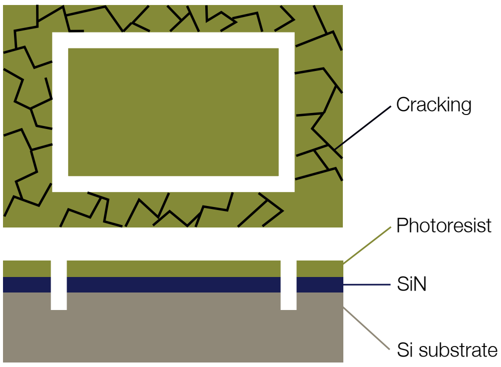

FIG. S7. Schematic describing the protecting outer design.

resonators area. The cracked resist patterns can then transfer into the underneath Si_3N_4 during etching, destroying the devices. A post-baking step of 15 minutes at 120 °C prior to the etching was found to be effective to improve the photoresist plasma resistance and to further to reduce the cracks on the surface, but not to completely remove them. We also investigated the effect of the carrier wafer thickness and the Si substrate thickness. Previous studies found in fact that photoresist cracking is initiated by wafer deformation caused by the helium backside cooling system and can be mitigated by employing a thicker substrate[24]. However, in our case, the photoresist cracking did not show any dependence on the substrate thickness.

We therefore surrounded the nanomechanical resonator's area of the device with a protecting outer ring (Fig. S7). This ring is etched into both the Si_3N_4 layer and the upper part of Si substrate in order to create a physical barrier. The resulting pattern is shown in S6b which clearly shows the effectiveness of the developed method to prevent any cracks from propagating toward the nanomechanical resonators. It is nevertheless important to notice that often photoresist cracking was confined to the outer area of the device without propagating toward the central area (S6c),

5. Beams collapsing

The centimeter-scale nanomechanical resonators are suspended by a fluorine based plasma etching performed at cryogenic temperature. The etching allows a quick and controllable release of the Si_3N_4 layer, without limitations from surface tension. However, plasma etching introduces charging effects due to the insulating nature of Si_3N_4. The charged Si_3N_4 can then be attracted by the Si substrate leading to the collapse of the nanoresonators.

Depending on the distance between the suspended resonator and the surrounding, we observed the Si_3N_4 being attracted toward or the side (Fig. S8c) or the bottom (Fig. S8b) of the opening around it. It follows that controlling the gap distance is an effective way to mitigate the collapse of the structures.

The distance from the wall along the in-plane direction can easily be controlled lithographically during the electron beam exposure. We observed that an opening larger than 70 µm is needed to eliminate the probability of sticking to the wall. This affects the exposed area and thus the total writing time, which in turn can increase the probability of stitching errors. However as discussed in section 3, a multi exposure with different resolutions is an effective solution to reduce the total writing time and eliminate stitching errors.

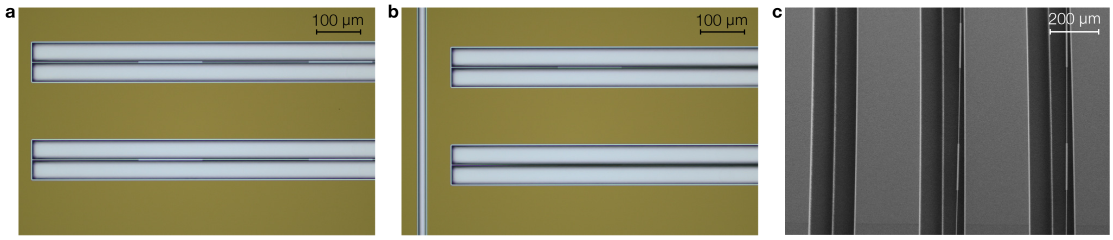

FIG. S8. **a**, Optical microscope picture of 3 cm nanomechanical resonators correctly suspended. **b**, Optical microscope picture of 3 cm nanomechanical resonators collapsed. **c**, Scanning electron microscope picture of 3 cm nanomechanical resonators sticking to the walls.

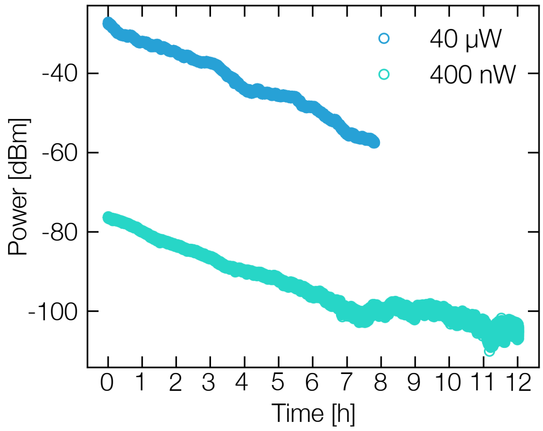

FIG. S9. Ringdown traces of the optimized nanoresonator excited at 214 kHz. The motion is probed with different laser powers in consecutive measurements, plotted in the same graph for comparison.

On the other hand, the distance between the suspended resonator and the Si substrate along the out-of-plane direction needs to be controlled by an etching step. To this end, we performed a cryo-DRIE of the Si substrate prior to the release step. A gap under the suspended Si_3N_4 larger than 50 µm was observed to be needed to avoid the collapse of the structure. The final pattern used in the main text has then a distance between the resonator and the wall of around 80 µm, while the gap under the Si_3N_4 after the cryo-DRIE is of 70 µm (Fig. S8a) .

6. Optothermal effects on the measured Q factor

The quality factor of the fabricated centimeter-scale resonators is experimentally measured with the ringdown method. The resonator is mechanically excited close to resonance with a sinusoidal function before turning off the excitation and measuring the decay time. A linear fit in the logarithmic scale of the observed decay allows us to extract the decay rate and thus the quality factor. It is however of paramount importance to avoid any unintentional excitation of the resonator during the measurement to extract the correct value of the quality factor.

One possible source of unintentional excitation is the laser employed to interferometrically probe the displacement of the resonator. The optical power impinging on the suspended resonator can in fact optothermally or optically drive it. To address this issue, we employed a laser operating at 1550 nm, the wavelength at which Si_3N_4 has negligible absorption. Moreover, we minimized the laser power coupling to the resonator down to 400 nW. This value is several orders of magnitude lower than the laser power conventionally used in previous studies (see for example[12]), where no effects correlated to optothermal or optical excitation were observed. To further dismiss any contributions we performed different ringdown measurements by varying the laser power incident on the resonator from 40 µW to 400 nW. The results in Fig. S9 show a comparable decay rate for all the traces, suggesting that optothermal or optical effects are indeed negligible. The measurements are performed on the optimized design reported in the main text in Fig. 4.

7. Multi-fidelity Bayesian optimization

The pursuit of efficient optimization of data scarce and high-fidelity black-box functions has led to Bayesian optimization techniques. These are statistical methods that yield a belief model over the entire domain $\mathscr{X}$ which is sequentially updated through newly acquired data. The generic Bayesian optimization (BO) algorithm was originally introduced by Jones *et al.*[25], and is a proxy-optimization scheme: instead of optimizing f directly over $\mathscr{X}$, one first selects a regressor R to model the response surface $\hat{f}(\mathscr{X})$ based on a design of experiments $\mathscr{D} = \{(x_1, f(x_1)), \dots, (x_n, f(x_n))\} \subset \mathscr{X}$, which is simply a set of n known input-output pairs. Importantly, the response surface also includes a measure of uncertainty on top of the predicted outcome. This is why BO is often performed with Gaussian process regression (GPR)[26–28]. Based on this regression model $R(\mathscr{D})$, a so-called acquisition function is built and optimized over the same domain. The goal of this proxy optimization is to suggest a new point x_{n+1} in $\mathscr{X}$ to be sampled with f, and as such, the design of experiments is augmented with $(x_{n+1}, f(x_{n+1}))$. In an algorithmic format, this can be expressed as follows:

Algorithm 1 Bayesian optimization

Require: Search space $\mathscr{X}$, regressor R, design of experiments $\mathscr{D}$, acquisition function acq, threshold condition C

while C is False **do**
  $\hat{f} \leftarrow R(\mathscr{D})$
  $x \leftarrow \text{argmax}_{x' \in \mathscr{X}} \text{acq}(x'; \hat{f})$
  $y \leftarrow f(x)$
  $\mathscr{D} \leftarrow \mathscr{D} \cup \{(x, y)\}$
end while

Given the scarcity of high-fidelity data, a standard solution is to acquire higher-throughput data with lower fidelity, i.e., with larger uncertainty with lower cost. This context of simultaneous high- and low-fidelity data structures, combined with the ideas of GPR, gives rise to a multi-fidelity data driven modelling paradigm. The first multi-fidelity GPR (MFGPR) method was introduced by Kennedy & O'Hagan[29], under the term cokriging. Numerous other MFGPR methods have been constructed and researched since then[30–32], reviewed by Liu *et al.*[33].

There are several ways in which Bayesian optimization (Algorithm 1) can be extended to handle regressors R over data sets with multiple fidelities, as is the case with MFGPR. A straightforward way can be described as follows, when the m indicates the function's fidelity:

Algorithm 2 Multi-fidelity Bayesian optimization

Require: Search space $\mathscr{X}$, fidelity space $\mathscr{Z}$, regressor R, DoE $\mathscr{D}$, acquisition function acq, threshold condition C

while C is False **do**
  $\hat{f} \leftarrow R(\mathscr{D})$
  $x, m \leftarrow \text{argmax}_{(x', m') \in \mathscr{X} \times \mathscr{Z}} \text{acq}(x', m'; \hat{f})$
  $y \leftarrow f_m(x, m)$
  $\mathscr{D} \leftarrow \mathscr{D} \cup \{(x, y)\}$
end while

Note that this algorithm updates Algorithm 1, so that the acquisition function samples from both the fidelity function.

Interfacing MFGPR with BO has been discussed in practice by Forrester *et al.*[34] and Huang *et al.*[35] While the former simply applied expected improvement (EI) acquisition on the prediction of a single-fidelity, the latter devised an augmented version of EI. This acquisition function multiplies the EI function applied to the highest fidelity, with fidelity-dependent parameters such as the ratio of computational cost and correlation between the highest fidelity and the fidelity in question. This effectively separates the design space and fidelity space aspects of the multi-fidelity problem. More recently, Jiang et al.[36] have applied a similar multiplicative factor principle to create variable-fidelity upper confidence bound (VFUCB).

To demonstrate the process outlined by Algorithm 2, we use cokriging as the regressor (R) along with VFUCB as the 2-fidelity ($\mathscr{Z} = \{\text{low}, \text{high}\}$) acquisition function (acq). See Fig. S10 for visualization in the case of multi-fidelity data $\mathscr{D}$ sampled from the high- and low-fidelity Forrester functions[34], a set of similar one-dimensional objective functions.

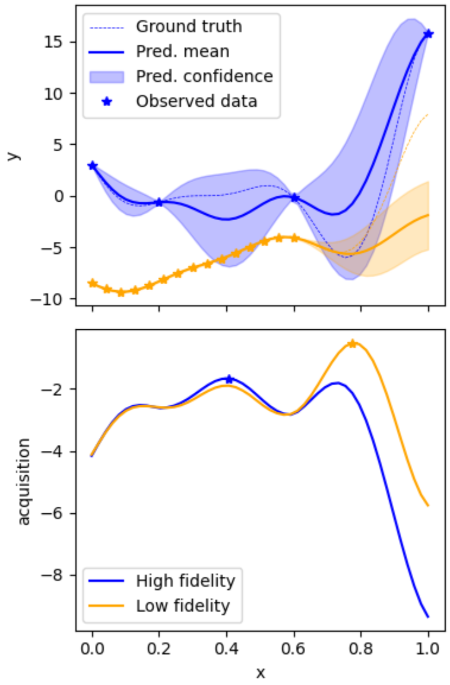

FIG. S10. (top) The input-output space of the objective function. The blue solid line indicates the cokriging predictive mean for the high-fidelity data (blue stars) sampled from the high-fidelity objective function (blue dashed line). The blue shaded area corresponds to the confidence interval (two predictive standard deviations). The orange counterparts show similar results for the low-fidelity scenario. (bottom) The input-acquisition value space. The blue and orange solid lines indicate the high- and low-fidelity branches of the VFUCB acquisition function respectively. The blue and orange stars respectively show the locations at which the high- and low-fidelity acquisition branches are maximized.

From Figure S10, the following can be inferred:

- The maximum acquisition value of the low-fidelity branch is higher than that of the high-fidelity acquisition. Therefore, the fidelity selection $m =$ high is made in step 3 of Algorithm 2.

- Compared to the maximizer of the high-fidelity acquisition branch, the maximizing x-value of the low-fidelity branch is closer to the minimizing x-value of the high-fidelity objective; the low-fidelity data is able to guide the optimization process.

8. Multi-fidelity Bayesian optimization initial random points dependency

Since our simulation-based design problem handles a stochastic optimization on the beam-like nanomechanical resonator, the initial design of experiments affects the convergence to the optimum solution. The resonator has a design space with nine design parameters, making the initial points (=25) affect the performance. Because we use multi-fidelity Bayesian optimization to reduce the total number of simulation evaluations, the curse of dimension is inevitable. **Figure S11** shows the optimization history when we start from four different sets of random initial points. The change of initial points affects the convergence speed as expected, along with different behavior in selecting the fidelity. However, the optimized design obtained for all cases has converged on a similar design by taking advantage of the multi-fidelity optimization.

9. PnC beam resonator's design parameters

The one-dimensional PnC resonator has a total length of 3 cm (or 3 mm) length with a total of 32 unit cells. During the Bayesian optimization, the quality factor of the resonator is maximized by changing nine design parameters. Figure S12 illustrates the design parameters of the optimized resonator discussed in the manuscript. Two parameters correspond to the defect's width (w_d) and length (L_d) in the bound of [0.5 μm, 3 μm], [150 μm, 1000 μm] ([15 μm, 100 μm]), respectively. Two parameters illustrate the width (r_w) and the length(r_L) ratio for each of the unit-cell in the bound of $[1.5, 3]$, $[0.25, 0.75]$, respectively. The

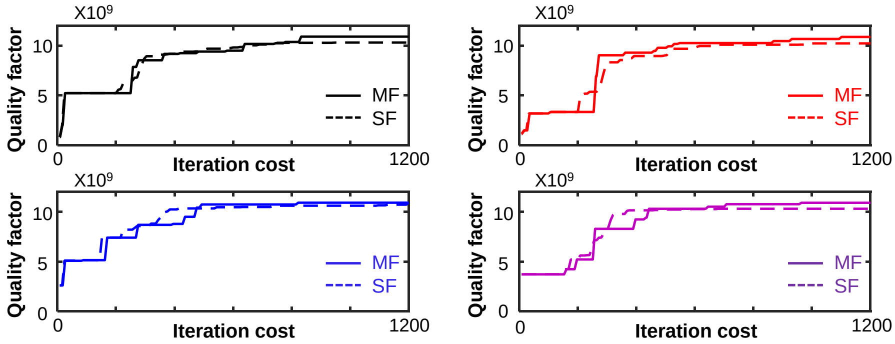

FIG. S11. Iteration history of the PnC beam nanomechanical resonator for optimizing Q_m with four different randomly selected initial points. The top-left is the iteration history discussed in the main text.

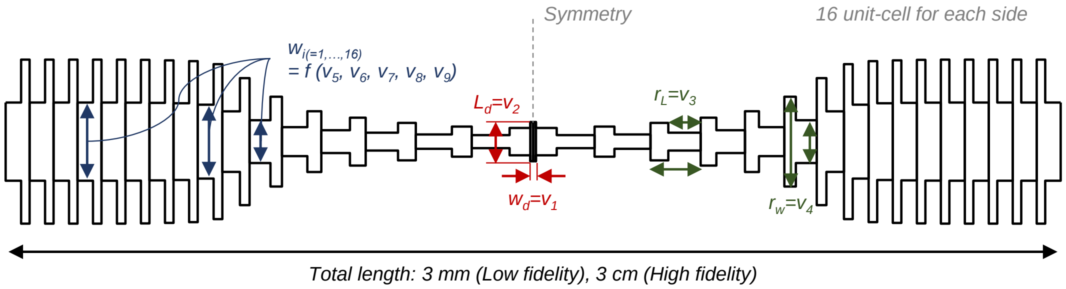

FIG. S12. Nanomechanical resonator model with 16 unit cells for each side and 9 design parameters. The total length is 3 cm (high-fidelity) and 3 mm (low-fidelity).

width ratio is the ratio between the wide and thin parts of the unit cells, and the length ratio is the ratio between the length of the thin part of the unit cell versus the total length of the unit cells. The tapered shape was defined by five design parameters in the bound of [0.5 µm, 3 µm]. We performed Piecewise Cubic Hermite Interpolating Polynomial for the 16 unit-cell's width of the thin part on the shape-determinating design parameters. The simulation was performed to find the maximum quality factor in the range of 100 kHz to 400 kHz, considering the defect mode using the bandgap. The length of the unit cells was determined considering the bandgap frequency matching condition, once the set of each unit cell's width is defined[5]. During the optimization, the resonator's thickness was set to 50 nm.

10. Quality factors of additional devices

This section presents the measurements conducted on additional fabricated devices, which share the same optimized design and are lithographically identical to the device featured in Fig. 4 of the main text. Figure S13 shows multiple ringdown traces from 3 devices. The uppermost blue curves correspond to four different measurements of the device discussed in the main text, while the green and light purple curves depict measurements obtained from two additional devices, each measured twice. Traces for the same device exhibit a comparable decay rate, resulting in the same quality factor, which is indicated above the first curve of each device. Linear fits for each trace are depicted as solid black lines.

The power values on the y-axis are adjusted relative to the maximum value of each curve before applying a 10 dBm offset between them. An additional 10 dBm spacing along the y-axis distinguishes different devices. Strong fluctuations resulting from unwanted temperature drifts and mechanical vibrations of the setup can push the measured signal outside the linear region of the interference signal for a brief time interval. This, in turn, leads to occasional spikes, as visible in some curves. The results demonstrate a consistent and reproducible quality factor among different measurements of each device. The observed variation among the quality factor of different devices suggests differences in surface quality among the devices, e.g., surface impurities and surface roughness[37], or different thicknesses. As further detailed in Sec. B, variation in the final thickness of each device might occur due to the difficulties in dissipating heat during the undercut process, significantly impacting the measured quality factor. Among all the devices fabricated and measured, we observed a variation in quality factor of around one order of

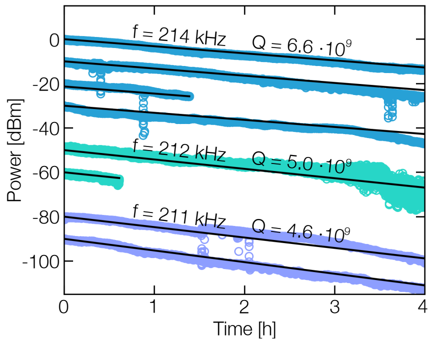

FIG. S13. Multiple ringdown traces of 3 fabricated nanoresonators with the optimized design presented in the main text. Traces corresponding to measurements from the same device are color-coded for easy identification. To facilitate comparison, each trace has been vertically shifted relative to its respective maximum value, $(P(t) - P_{max})$. Subsequently, an incremental 10 dBm offset has been applied to each trace, starting with the top-most blue trace. An additional 10 dBm offset is applied between measurements from different devices.

magnitude, which is in line with typical variations found in other works.

11. Ultra High Vacuum Setup

We developed a UHV setup designed to operate at pressures close to 10^{-9} mbar, aiming to minimize gas damping and measure the intrinsic quality factor of the fabricated centimeter-scale resonators. This pressure value approaches the final pressure of the employed turbomolecular pump, necessitating setup optimization to reduce the volume and the surface of the overall vacuum system, and potential outgassing sources. This section provides details on the vacuum setup.

The vacuum setup consists of a cylindrical chamber (Fig. S14a) with 8 CF-40 flanges along the lateral surface and 2 CF-100 flanges on the top and the bottom. The CF-40 flanges are employed for electrical and optical connection and the vacuum gauge. The bottom flange directly connects to a turbo molecular pump via a valve and a large opening CF-63 (Fig. S14 b) without any restrictive hoses that could hinder pumping efficiency. This allows the volume and the surface of the overall vacuum system to be reduced, improving the pumping efficiency. The chamber incorporates UHV-compatible components for sample manipulation, including a triaxial nanopositioner, sample holders, and piezoelectric plates soldered with lead-free solder to KAPTON wires. Lacking a load lock, we fully open the chamber for each new sample through the top viewport and do not require chamber baking. After loading a sample, we typically purge the chamber with nitrogen three times while the backing pump is running before starting the turbo molecular pump.

It is crucial to note that the employed pressure gauge's operational range spans from 10^{-9} to 10^{3} mbar, whereas the turbo-molecular pump's final pressure is 5×10^{-10} mbar. Consequently, the pressure gauge provides only a lower estimate of the vacuum level.

a

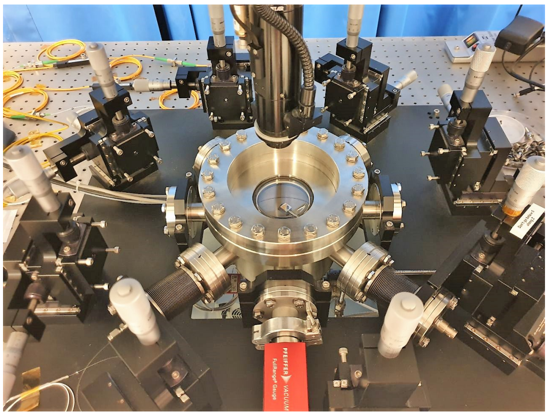

b

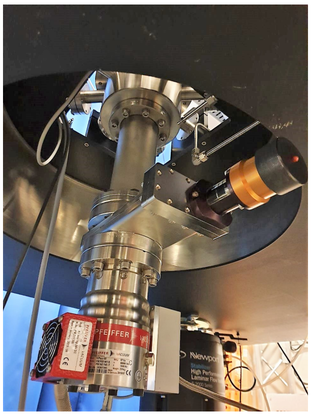

FIG. S14. Vacuum chamber and turbo molecular pump. **a**, Photograph of the vacuum chamber with the pressure gauge mounted at the vacuum chamber. **b**, Photograph of the turbo molecular pump connected to the bottom of the vacuum chamber.

[1] C. Reinhardt, T. Müller, A. Bourassa, and J. C. Sankey, Physical Review X **6**, 021001 (2016).
[2] R. A. Norte, J. P. Moura, and S. Gröblacher, Physical Review Letters **116**, 10.1103/PhysRevLett.116.147202 (2016).
[3] A. H. Ghadimi, D. J. Wilson, and T. J. Kippenberg, Nano letters **17**, 3501 (2017).
[4] Y. Tsaturyan, A. Barg, E. S. Polzik, and A. Schliesser, Nature Nanotechnology **12**, 776 (2017).
[5] A. H. Ghadimi, S. A. Fedorov, N. J. Engelsen, M. J. Bereyhi, R. Schilling, D. J. Wilson, and T. J. Kippenberg, Science **360**, 764 (2018).
[6] M. Rossi, D. Mason, J. Chen, Y. Tsaturyan, and A. Schliesser, Nature **563**, 53 (2018).
[7] E. Serra, B. Morana, A. Borrielli, F. Marin, G. Pandraud, A. Pontin, G. A. Prodi, P. M. Sarro, and M. Bonaldi, Journal of Microelectromechanical Systems **27**, 1193 (2018).
[8] C. Reetz, R. Fischer, G. Assumpção, D. McNally, P. Burns, J. Sankey, and C. Regal, Physical Review Applied **12**, 044027 (2019).
[9] J. Guo, R. Norte, and S. Gröblacher, Physical Review Letters **123**, 223602 (2019).
[10] C. M. Pluchar, A. R. Agrawal, E. Schenk, D. J. Wilson, and D. J. Wilson, Applied Optics **59**, G107 (2020).
[11] D. Høj, F. Wang, W. Gao, U. B. Hoff, O. Sigmund, and U. L. Andersen, Nature Communications **12**, 5766 (2021).
[12] D. Shin, A. Cupertino, M. H. J. de Jong, P. G. Steeneken, M. A. Bessa, and R. A. Norte, Advanced Materials **34**, 2106248 (2022).
[13] M. J. Bereyhi, A. Arabmoheghi, A. Beccari, S. A. Fedorov, G. Huang, T. J. Kippenberg, and N. J. Engelsen, Physical Review X **12**, 021036 (2022).
[14] M. J. Bereyhi, A. Beccari, R. Groth, S. A. Fedorov, A. Arabmoheghi, T. J. Kippenberg, and N. J. Engelsen, Nature Communications **13**, 3097 (2022).
[15] T. Gisler, M. Helal, D. Sabonis, U. Grob, M. Héritier, C. L. Degen, A. H. Ghadimi, and A. Eichler, Physical Review Letters **129**, 104301 (2022).
[16] J. R. Pratt, A. R. Agrawal, C. A. Condos, C. M. Pluchar, S. Schlamminger, and D. J. Wilson, Physical Review X **13**, 011018 (2023).
[17] J. Guo, J. Chang, X. Yao, and S. Gröblacher, Nature Communications **14**, 4721 (2023).
[18] L. Dania, D. S. Bykov, F. Goschin, M. Teller, and T. E. Northup, Ultra-high quality factor of a levitated nanomechanical oscillator (2023), arxiv:2304.02408 [quant-ph].
[19] S. Schmid, L. G. Villanueva, and M. L. Roukes, *Fundamentals of Nanomechanical Resonators* (Springer International Publishing, Cham, 2023).
[20] S. S. Verbridge, R. Ilic, H. G. Craighead, and J. M. Parpia, Applied Physics Letters **93**, 013101 (2008).
[21] S. A. Saarinen, N. Kralj, E. C. Langman, Y. Tsaturyan, and A. Schliesser, Optica **10**, 364 (2023).
[22] D. J. Dougherty, R. E. Muller, P. D. Maker, and S. Forouhar, Journal of Lightwave Technology **19**, 1527 (2001).
[23] M. D. Henry, C. Welch, and A. Scherer, Journal of Vacuum Science & Technology A **27**, 1211 (2009).
[24] L. Sainiemi and S. Franssila, Journal of Vacuum Science & Technology B: Microelectronics and Nanometer Structures Processing, Measurement, and Phenomena **25**, 801 (2007).
[25] D. R. Jones, M. Schonlau, and W. J. Welch, Journal of Global optimization **13**, 455 (1998).
[26] P. I. Frazier, arXiv preprint arXiv:1807.02811 (2018).
[27] J. Snoek, H. Larochelle, and R. P. Adams, in *Advances in Neural Information Processing Systems 25*, edited by F. Pereira, C. J. C. Burges, L. Bottou, and K. Q. Weinberger (Curran Associates, Inc., 2012) pp. 2951–2959.
[28] B. Shahriari, K. Swersky, Z. Wang, R. P. Adams, and N. De Freitas, Proceedings of the IEEE **104**, 148 (2015).
[29] M. C. Kennedy and A. O'Hagan, Biometrika **87**, 1 (2000).
[30] Z.-H. Han and S. Görtz, AIAA journal **50**, 1885 (2012).
[31] L. Le Gratiet and J. Garnier, International Journal for Uncertainty Quantification **4** (2014).
[32] C. Williams, E. V. Bonilla, and K. M. Chai, Advances in neural information processing systems , 153 (2007).
[33] H. Liu, J. Cai, and Y.-S. Ong, Knowledge-Based Systems **144**, 102 (2018).
[34] A. I. Forrester, A. Sóbester, and A. J. Keane, Proceedings of the royal society a: mathematical, physical and engineering sciences **463**, 3251 (2007).
[35] D. Huang, T. T. Allen, W. I. Notz, and R. A. Miller, Structural and Multidisciplinary Optimization **32**, 369 (2006).
[36] P. Jiang, J. Cheng, Q. Zhou, L. Shu, and J. Hu, AIAA Journal **57**, 5416 (2019).
[37] L. G. Villanueva and S. Schmid, Physical Review Letters **113**, 227201 (2014).
