# Supplementary material for: Centimeter-scale nanomechanical resonators with low dissipation
Source: Nat Commun. 2024 May 18;15:4255. doi: 10.1038/s41467-024-48183-7 (PMC11102468; doi:10.1038/s41467-024-48183-7)
Supplement: Supplementary file 3 — Description of Additional Supplementary Files [file 41467_2024_48183_MOESM3_ESM.pdf]

Description of Additional Supplementary Files

Supplementary Video:

The supplementary video visualizes the evolution of multi-fidelity Bayesian optimization. The results were postprocessed after the optimization. The left panel illustrates the geometry of simulated highfidelity (HF) and low-fidelity (LF) phononic crystal designs. The HF model changes eight times slower than its LF counterpart, which is an adjustment for iteration cost during the optimization. At the top, each fidelity count is tracked along with the total iteration cost, and the objective (corresponding to the maximum quality factor) is updated with the current best HF design. On the right, three graphs depict the simulated design's quality factor and distance from the previous argmax point. The multifidelity Bayesian optimization kicks off with 25 random samples for each fidelity. The final optimized design here was manufactured as the work's optimum design.
